# Reporting stAndards for research in PedIatric Dentistry (RAPID): an expert consensus-based statement

**DOI:** 10.1186/s12903-021-01698-7

**Published:** 2021-07-23

**Authors:** Jayakumar Jayaraman, Vineet Dhar, Kevin J. Donly, Ekta Priya, Daniela P. Raggio, Noel K. Childers, Timothy J. Wright, Venkateshbabu Nagendrababu, Mike Clarke, Nigel King, Jan Clarkson, Nicola P. T. Innes

**Affiliations:** 1 Department of Pediatric Dentistry, Virginia Commonwealth University School of Dentistry, 520 North 12th Street, Richmond, VA 23298 USA; 2 University of Maryland School of Dentistry, Baltimore, MD 21201 USA; 3 University of Texas Health School of Dentistry, San Antonio, TX 78229 USA; 4 Division of Children’s Dentistry and Orthodontics, School of Dentistry, International Medical University, Kuala Lumpur, Malaysia; 5 Faculdade de Odontologia, Universidade de Sao Paulo, Sao Paulo, Brazil; 6 School of Dentistry, University of Alabama, Birmingham, AL 35233 USA; 7 University of North Carolina At Chapel Hill, Chapel Hill, NC 27514 USA; 8 College of Dental Medicine , University of Sharjah, Sharjah, United Arab Emirates; 9 Queen’s University Belfast, Belfast, BT7 1NN UK; 10 University of Western Australia, Perth, WA 6009 Australia; 11 University of Dundee, Dundee, DD1 4HR UK; 12 School of Dentistry , Cardiff, CF14 4XY Wales UK

**Keywords:** RAPID, Pediatric dentistry, Children, Reporting, Guidelines, Delphi

## Abstract

**Background:**

Reporting guidelines for different study designs are currently available to report studies with accuracy and transparency. There is a need to develop supplementary guideline items that are specific to areas within Pediatric Dentistry. This study aims to develop Reporting stAndards for research in PedIatric Dentistry (RAPID) guidelines using a pre-defined expert consensus-based Delphi process.

**Methods:**

The development of the RAPID guidelines was based on the Guidance for Developers of Health Research Reporting Guidelines. Following a comprehensive search of the literature, the Executive Group identified ten themes in Pediatric Dentistry and compiled a draft checklist of items under each theme. The themes were categorized as: General, Oral Medicine, Pathology and Radiology, Children with Special Health Care Needs, Sedation and Hospital Dentistry, Behavior Guidance, Dental Caries, Preventive and Restorative Dentistry, Pulp Therapy, Traumatology, and Interceptive Orthodontics. A RAPID Delphi Group (RDG) was formed comprising of 69 members from 15 countries across six continents. Items were scored using a 9-point rating Likert scale. Items achieving a score of seven and above, marked by at least 70% of RDG members were accepted into the RAPID checklist items. Weighted mean scores were calculated for each item. Statistical significance was set at p < 0.05 and one-way ANOVA was used to calculate the difference in the weighted mean scores between the themes.

**Results:**

The final RAPID checklist comprised of 128 items that were finalized and approved by the RDG members in the online consensus meeting. The percentage for high scores (scores 7 to 9) ranged from 69.57 to 100% for individual items. The overall weighted mean score of the final items ranged from 7.51 to 8.28 (out of 9) and the difference was statistically significant between the themes (p < 0.05).

**Conclusions:**

The RAPID statement provides guidance to researchers, authors, reviewers and editors, to ensure that all elements relevant to particular studies are adequately reported.

**Supplementary Information:**

The online version contains supplementary material available at 10.1186/s12903-021-01698-7.

## Background

Evidence-based practice (EBP) is recognized as a critical foundation element for providing safe and effective healthcare. EBP relies on judicious integration of good scientific evidence, clinical expertise, and individual patient needs and preferences [1]. A good quality research manuscript should contain all the relevant details and vital information as it is an important source of knowledge in healthcare education and clinical research [2]. To enable decision making in clinical practice, the research has to be reported clearly, transparently, and provide sufficient information to the readers [3]. In contrast, incomplete reporting can prevent the replication of studies, adversely affect scientific progress, lead to misinterpretation and potentially result in inappropriate clinical application [4]. Data suggests that the reporting quality of biomedical research is suboptimal [5]. A reporting guideline is a simple list of information and a structured tool for investigators to use while drafting their manuscripts [6]. In oral health research, guidelines have assisted researchers in developing and writing manuscripts by improving the overall completeness and transparency of the reports [7].

Although journals in dentistry have endorsed various reporting guidelines specific to different study designs, for example, CONSORT for randomised clinical trials, STROBE for observational studies, and PRISMA for systematic reviews, it has been shown that studies are still being published with low reporting quality [8]. The reason for poor reporting could be a lack of knowledge of the existing reporting guidelines, inappropriateness of items, or inability to adapt them from the respective reporting guidelines by authors, reviewers, and journal editors. As in other specialities of healthcare, manuscripts published in Pediatric Dentistry vary in quality and are thus subject to risk of bias, lack of clarity and poor transparency. A study that evaluated 173 randomised trials published in Pediatric Dentistry found that the overall quality was “poor” with varied heterogeneity amongst published trials [9]. Similarly, evaluation of the quality of systematic reviews in Pediatric Dentistry found inadequacies in the reporting and identified areas for improvement [10]. In Pediatric Dentistry, various important information relevant to children's oral health has not been adequately reported [11]. It has become evident that there is a need for the development of supplementary guidelines specific to areas within Pediatric Dentistry due to the importance of growth and development of the pediatric population, as well as pediatric targeted interventions [12]. This includes, but is not limited to, caries risk assessment, parental oral health literacy, and behavior rating. These factors are considered important to improve the readers’ understanding of study findings in Pediatric Dentistry. The ‘Reporting stAndards for research in PedIatric Dentistry’ (RAPID) group has been formed to develop reporting guidelines specifically for Pediatric Dentistry journals. The RAPID reporting guidelines are aimed at improving the quality of research reporting in Pediatric Dentistry and thereby benefit researchers, clinicians, patients, and other stakeholders involved in the care and well‐being of children. The aim of this paper was to report the development of the RAPID guidelines using a pre-defined expert consensus-based Delphi process.

## Methods

**Fig. 1:**
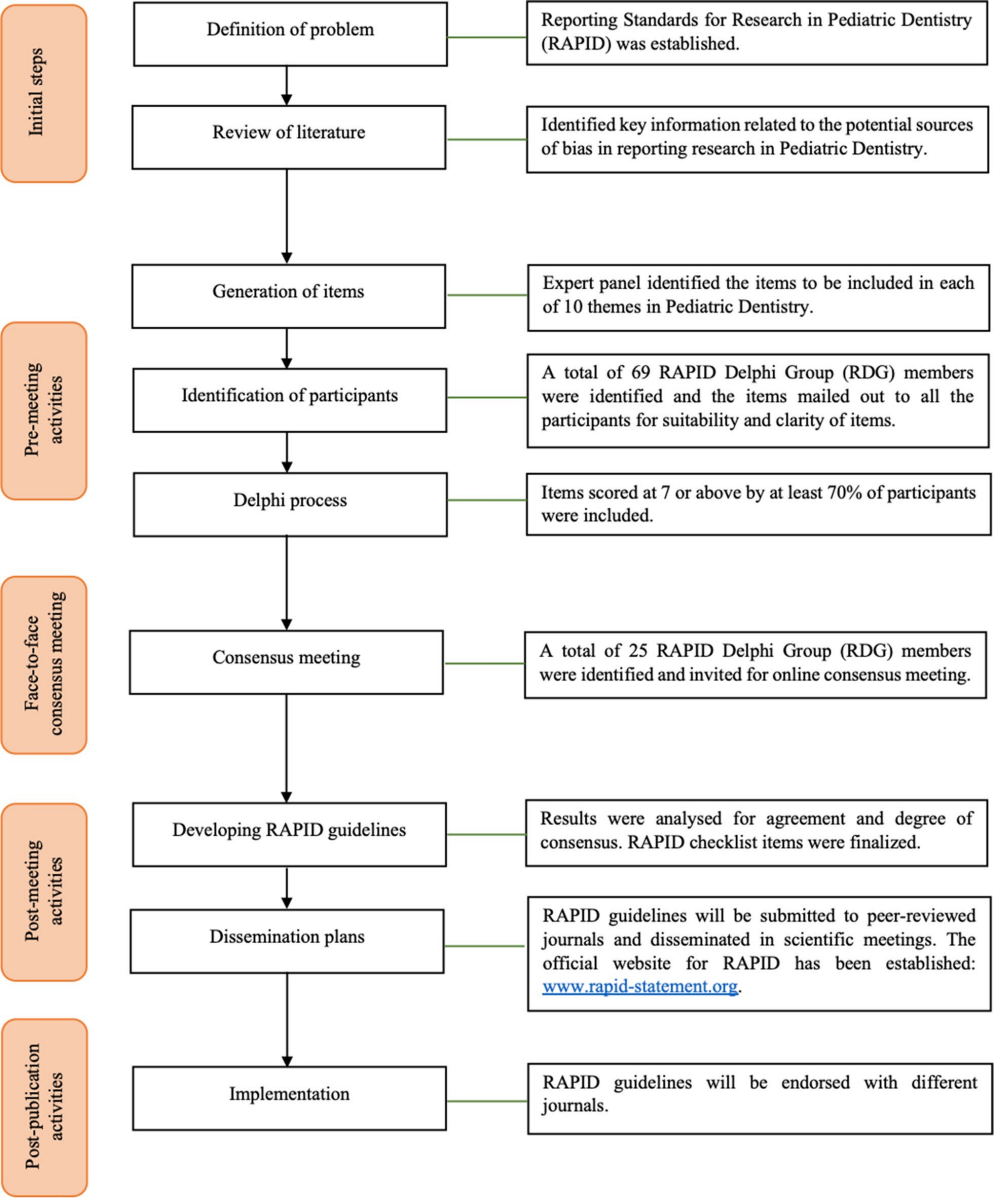
Flowchart of the RAPID consensus development process

The development of the RAPID guidelines was based on the Guidance for Developers of Health Research Reporting Guidelines [13]. A flowchart of the RAPID consensus development process is presented in Fig. 1. In addition, this project employed a five-phase process including a Delphi study in accordance with guidance on conducting and reporting Delphi study (CREDES) [14].

### Establishing the Executive Group

The Executive Group (EG) was formed by the project leader (JJ) and co-leaders (VD, KD) to develop the RAPID project. The members of the EG were selected based on their scientific and clinical experience and comprised of Pediatric Dentists and experts involved in guideline development process. The EG members (EP, JC, DPR, NC, TW, NK, VN, MC, NPTI) were represented from different institutions across diverse geographical locations.

### Developing initial RAPID checklist items

**Fig. 2:**
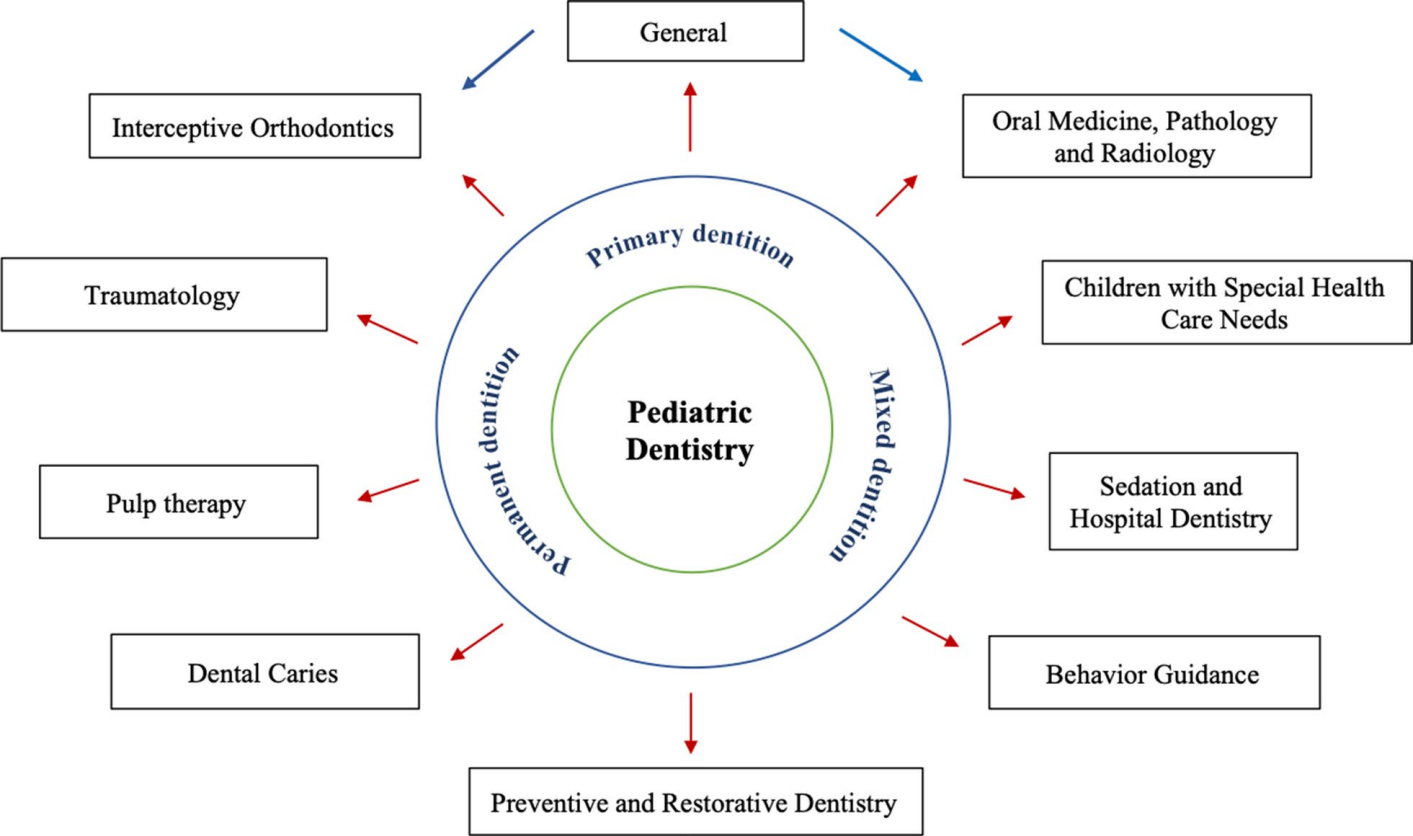
Themes in Pediatric Dentistry included in the RAPID checklist

A comprehensive search of the literature found that no reporting guideline specific to Pediatric Dentistry existed. The EG identified ten themes and draft checklist items were developed under each theme. The themes were categorized as: General, Oral Medicine, Pathology and Radiology, Children with Special Health Care Needs, Sedation and Hospital Dentistry, Behaviour Guidance, Dental Caries, Preventive and Restorative Dentistry, Pulp Therapy, Traumatology, and Interceptive Orthodontics (Fig. 2). Following critical appraisal of the literature, the project leader (JJ) and co-leaders (VD, KD) developed draft checklist items under each theme and all the items were vetted by the EG.

### Delphi process

The project leaders and EG formed the RAPID Delphi Group (RDG) and, based on the recommendation of the EG members, 80 members were initially selected to participate in the Online Delphi Process. An e-mail invitation, sent to all potential participants, contained information on the scope of the RAPID project and the expectations of the RDG members in the Delphi process. Based on the responses, 69 members comprising 30 Academicians, 12 practicing Pediatric Dentists, four Epidemiologists, four General Dentists, four Journal Editors, two Clinical Trialists, four Dental Public Health Specialists, three Health Economists, two Pediatricians, two Dental Nurses, and two Parents were included in the online Delphi study. The RDG members represented 15 countries across six continents. The criteria for selection of RDG members under each category has been described briefly in the RAPID development protocol publication [15].

A document containing details of the RAPID themes, checklist items, criteria for scoring, and link to the survey was sent to all RDG members. The survey was conducted via SurveyMonkey (SVMK Inc, San Mateo, CA). For each item, the members were asked to score using a 9-point rating Likert scale (1 = strongly disagree; 2 mostly disagree; 3 = somewhat disagree; 4 = slightly disagree; 5 = neutral; 6 = slightly agree; 7 = somewhat agree; 8 = mostly agree; 9 = strongly agree) [16]. In addition, the members were asked to provide suggestions for improvement of the checklist items in the “Comments” box under each item. Items achieving a score of 7 or above (7 = somewhat agree, 8 = mostly agree, 9 = strongly agree) on the 9-point rating Likert scale from at least 70% of the RDG members were eligible for inclusion in the RAPID checklist. Members were given three weeks to respond to the survey with reminder e-mails sent one week, and two days before the deadline. All 69 members responded to the survey. In the 9-point rating Likert scale, items scoring between 7 and 9 alone were included in the analysis. Items scoring 7 or above from at least 70% of the participants were deemed eligible for inclusion in the RAPID checklist after the first round, whereas items that scored 7 and above by 30% to 70% of the participants that were revised based on reviewer comments and included in the second round of the Delphi process. Any item that was scored between 7 and 9 by less than 30% of the participants was excluded from the checklist. On completion of the first round, a summary of the outcomes, scores achieved for each item, and the revised items were shared with the RDG members. The second round of the Delphi process followed the same methodological process as that of the first round. In total, 62 members completed the Round 2 Delphi. The items scored in both Round 1 and Round 2 Delphi rounds were analyzed and included in the final RAPID statement.

### Online consensus meeting

As per protocol [15], the EG were to conduct a face-to-face consensus meeting to discuss the items included in the final RAPID statement. However, due to the COVID-19 pandemic, the Executive Group agreed to change this to a virtual meeting using Zoom (Zoom Video Communications Inc, San Jose, CA) video conferencing platform. For the purpose of validity, only the members who participated in the online Delphi process were included in the face-to-face consensus meeting. From the RDG group, 35 members were randomly selected, and were invited by e-mail to participate in the meeting, out of which 25 agreed to participate. Information documents containing the date, time, agenda and link for the meeting, final checklist items, and data analytics for each item was sent to the participants two weeks prior to the scheduled meeting. Members were invited to send their comments on the items to allow discussion at the online meeting. The Zoom meeting took place in September 2020 and was attended by 28 members including 12 Academicians, 8 Pediatric Dentists, 3 Journal Editors, 1 Dental Nurse, 1 Epidemiologist, 1 Clinical Trialist, 1 General Dentist, and 1 Parent. The meeting was chaired by the project leaders (JJ, VD, KD) who presented the rationale for the RAPID guidelines, and discussed the scores obtained in the Round 1 and Round 2 Delphi including the items that achieved the highest and the lowest scores in the Delphi rounds. In addition to the scores, the knowledge translation strategy for the RAPID project was presented to members. Following this, members discussed the contents in the RAPID items and any suggestions that might improve the clarity of the items. The online consensus meeting lasted for 65 minutes. It was recorded and the minutes were taken for future reference.

### Analysis of the scored items

The descriptive statistics for the distribution of percentage scores was derived for each item based on the response received from the Round 2 Delphi. The cumulative percentage was calculated based on three criteria: Low (Scores 1 to 3), Moderate (Scores 4 to 6), and High (Scores 7 to 9). Items scored between 7 and 9 by over 70% of participants were included in the final checklist. The weighted mean was calculated for each item based on the individual score (0 to 9) and the number of respondents who scored the item. Following this, an overall weighted mean was determined for items in each theme. In addition, to understand the distribution of the scores, the median (Q2) and inter‐quartile range (Q1–Q3) of the item scores of 7 and above were calculated. Statistical significance was set at alpha level of 0.05 and one-way ANOVA was used to calculate the difference observed in the weighted mean scores between the themes (SPSS Version 20.0, SPSS IBM Inc, Armonk, NY).

## Results

### Finalized items through RAPID Delphi Process

In total, 128 items were finalized and approved by the RDG members at the end of Round 2 of the Delphi process. The finalized themes and the number of items under each theme (in parenthesis) are presented: General (28), Oral Medicine, Pathology and Radiology (10), Children with Special Healthcare Needs (8), Sedation and Hospital Dentistry (10), Behavior Guidance (10), Dental Caries (13), Preventive and Restorative Dentistry (15), Pulp Therapy (13), Trauma (12), and Interceptive Orthodontics (9). The items within the theme were categorized into the following topics: Patient Information, Unit of Analysis, Intervention, Moderators, Assessment, and Outcomes. The “General” theme covered items relevant to reporting research and general topics in Pediatric Dentistry, whilst the other themes contained items specific to the specialty or area of interest (Tables 1, 2, 3, 4, 5, 6, 7, 8, 9 and 10).

**Table 1:** Checklist items in the “General” theme

Topic	Item number	Checklist items	Reported on page number
Reporting Research	1	Indicate that the study adhered to reporting guidelines for main study types (or appropriate extensions where one exists). For example, Case Report – CARE, Observational study—STROBE, Clinical trial- CONSORT, Systematic review—PRISMA etc	
	2	Report the period during which the study was conducted, for both observational and experimental studies	
	3	Include “children's dentistry” or “pediatric dentistry” or “paediatric dentistry” as keywords	
	4	Report details of the Institutional Review Board including the approval number	
	5	Include information on child specific factors that may influence sample size calculation (drop out ratio, cluster effect etc.)	
	6	Include information on the statistical analysis used in the study, including the statistical package	
	7	Include information on how the examiners were calibrated prior to the assessment	
	8	Report the examiner reliability scores (intra-examiner and/or inter-examiner scores) and report discrepancy in the scores, if any	
	9	Report both statistically significant and non-significant outcomes in the results, tables and figures with proper effect measures and variation data (confidence interval etc.)	
	10	Report the impact of the intervention on oral health related quality of life, where relevant	
	11	For intervention studies with multiple interventions, include information on whether a process evaluation was undertaken (staff training, service provided etc.)	
Patient Information	12	Include information on age, sex, Body Mass Index (BMI), and overall health status	
	13	Include information on informed consent/assent for children and adolescents based on national/regional/local regulations and on the relationship from whom the consent was obtained	
	14	Include information on the community of interest and social, psychological determinants of health (SES, ethnicity, Immigration status etc.)	
	15	Include information on the behavior of the child included in the study using a validated behavior rating scale, where relevant	
	16	Include information on extra-oral and intra-oral findings relevant to the focus of the study, where relevant	
Intervention	17	Include information on all the materials, instruments, software, and equipment relevant to the focus of the study	
	18	If a new treatment is investigated, also include information on the recommended "gold standard" treatment	
	19	If multiple treatments are required, include information on the sequence of treatment	
Moderators	20	Include information on any challenges (child’s/parent’s/operator’s level) encountered during dental treatment and care delivery pathway, where relevant	
	21	Include information on contraindication to any dental materials or dental treatment, where relevant	
	22	Include information on any unanticipated events or consequences of the treatment rendered	
	23	Include information on the anticipatory guidance provided to the child/carer, where relevant	
	24	Include information on the level of expertise of the treatment provider and its influence on overall outcome, where relevant	
Outcomes	25	Include information on the child based and parent/carer-based outcomes, where relevant	
	26	Include information on the use of pediatric-specific standardized tools to assess the observations and outcomes (Caries, Gingiva, Plaque, Periodontal health etc.), where relevant	
	27	Report the outcomes specific to the age of the child (in chronological years, physiological or developmental milestones), where relevant	
	28	Include information on the follow up intervals for both clinical and radiographic outcomes at appropriate time intervals, where relevant

**Table 2:** Checklist items in the “Oral Medicine, Pathology and Radiology” theme

Topic	Item number	Checklist items	Reported on page number
Patient information	1	Include information on the family and social history relevant to the syndrome or condition	
	2	Report the prominent extra-oral and intra-oral features specific to the syndrome	
	3	Include information on how the consent was obtained for using the tooth samples along with indication for extraction, except for de-identified samples	
Intervention	4	Include information on how the teeth or biopsy specimens were disinfected, stored or transported prior to use	
	5	Include information on the laboratory process of the testing method and include the commercial details of all the materials used in the testing	
	6	Include information on any special consideration taken to manage the behavior of the patient in relation to the condition	
	7	Report if any special precaution is required for dental management for the patient relevant to the condition	
Outcome	8	Include information on other similar conditions and how it varies from the condition reported	
	9	Report in the text, the most salient feature or area of importance of the reported image	
	10	Provide the salient features in the legend of the reported image

**Table 3:** Checklist items in the “Children with Special Health Care Needs” theme

Topic	Item number	Checklist items	Reported on page number
Patient information	1	Report the medical condition, where relevant	
	2	Include information on the birth, family, genetic and social history of hereditary etiology, where relevant	
	3	Report any considerations in the assessment of pediatric airway for sedation or general anesthesia where appropriate	
Intervention	4	Include information on any modifications in dose calculations (pre-medications, analgesia, anesthesia etc.), where relevant	
	5	Report any consultation with child’s physician or any referrals	
	6	Report significant findings of report obtained from the patient’s physician	
	7	Report any modifications made for safe delivery of dental care, any pre-operative or post-operative care, medication list, allergies, etc	
	8	Indicate how communication was established with the child and any modifications to the behavior guidance technique, where relevant

**Table 4:** Checklist items in the “Sedation and Hospital Dentistry” theme

Topic	Item number	Checklist items	Reported on page number
Patient information	1	For surgical procedures under sedation or general anesthesia, include information on pre-operative evaluation checklist including vital signs, airway evaluation, tonsillar size, and nil per os (NPO) status	
	2	Include information on the syndrome from historical and genetic standpoint	
	3	Include information on the justification for considering treatment under sedation or general anesthesia	
	4	Include information on consent, risks and benefits for treatment under sedation or general anesthesia	
Intervention	5	Report how the drug dose was calculated and the mode of delivery	
	6	Report local anesthetic administered, type, dosage, with or without vasoconstrictor	
	7	Include information on the anesthesia protocol including intubation, induction and monitoring	
	8	Include information on the medications given for pain management	
Outcome	9	Include information on the post-operative instructions and recovery	
	10	Report any intra-operative or post-operative complications

**Table 5:** Checklist items in the “Behavior Guidance” theme

Topic	Item number	Checklist items	Reported on page number
Intervention	1	Include information on the type of behavior guidance technique provided to the parent/caregiver	
	2	Include information on the indications, risks, benefits, and alternatives of the behavior guidance technique of interest	
Assessment/Outcome	3	Include information on the people involved in behavior guidance (dentist, parent, nurse) and their role (active, passive) during delivery of the behavior guidance	
	4	Include information on the settings for behavior management-private dental setting, public hospital, specialist pediatric dental setting, general dental setting	
	5	Report parent’s/caregiver's and child’s perspectives on behavior guidance technique used, where relevant	
	6	Report dentist's, parent's preferences and child’s preferences (wherever appropriate) of behavior guidance	
	7	Report the experience or behavior of the child of previous dental visit, if applicable	
	8	Report the influence of the behavior guidance technique used or studied on the intervention, where relevant	
	9	Report the dental anxiety, fear and/or behavior of the child included in the study using validated measures (Examples: Facial Image Scale, Dental subscale of children's fear survey schedule, Frankl’s behavior rating scale)	
	10	Report any challenges (child’s/parent’s/operator’s level) encountered during dental treatment and care delivery pathway

**Table 6:** Checklist items in the “Dental Caries” theme

Topic	Item number	Checklist items	Reported on page number
Patient information/unit of analysis	1	Include information on the method of reporting dental caries and the criteria used (e.g. DMFT, DEFT, Prevalence rate etc.)	
	2	Include information on age and population specific use of terminologies (e.g. Early Childhood Caries)	
	3	Report classification of clinical and radiographical caries based on a standardized classification system (e.g. ICDAS etc.), where relevant	
	4	Report the unit of analyses (e.g. the child, the tooth). Include information on how the analyses were performed considering possible clustering (e.g. more than one tooth per child, more than one child per school etc.)	
	5	Include information on risk factors such as the dietary and oral hygiene practices of the children included in the report, where relevant	
Intervention/moderators	6	Include information on the method used for caries detection (e.g. Visual, radiographs, laser etc.)	
	7	Report the category of risk using a caries risk assessment tool, where relevant	
	8	Report the influence of caries risk on the treatment outcomes, where relevant	
	9	Report individual and tooth-related factors influencing the intervention	
	10	Include information on the rationale for management of dental caries based on standardized management system (e.g. ICCMS etc.), where relevant	
	11	Include information on primary, secondary and tertiary levels of prevention strategies used in the study, where relevant	
	12	Include information on topical and systemic fluoride intake including water fluoride level, if available	
	13	Report compliance with interventions and quality of self-reported pre-study information (completeness of baseline diet history etc.)

**Table 7:** Checklist items in the “Preventive and Restorative Dentistry” theme

Topic	Item number	Checklist items	Reported on page number
Intervention/moderators	1	Include information on the dosage, vehicle, and regimen followed for any in-office preventive strategies used (xylitol, professional fluoride etc.)	
	2	Include information on material type, technique, follow-up protocols, and method used to evaluate effectiveness of preventive treatment	
	3	Include information on diet and biofilm control measures (toothbrush, interdental aids, antimicrobial strategies) used	
	4	Include information on the indications, risks, and benefits of the restorative material/technique of interest in pediatric population	
	5	Include information on the difficulty/ease of recruiting children/samples for the study using the restorative material/technique of interest	
	6	Include information on the carious tissue removal (selective/non-selective/stepwise) or non-removal process	
	7	Report the justification for use of restorative material or technique in primary and permanent teeth, i.e. to highlight the difference or uniqueness in primary teeth/young permanent teeth or permanent teeth	
	8	Include information on the level of technique sensitivity or skills/experience required (for example, if a technique can be easily be performed by novice graduates/general dental practitioners or needs specialists)	
	9	Report the influence of caries risk and patient characteristics on the treatment outcomes	
	10	Report whether moisture control was maintained and what was used for moisture control (rubber dam, cotton rolls, suction devices etc.) during the restorative procedure	
	11	Report the socioeconomic status of the population and the type of service where the study was conducted (for example, public health vs private practice; third party insurance vs private pay vs federal insurance), where relevant	
Assessment/outcomes	12	Include information on whether the restorative/preventive regimen followed established protocols (IAPD, AAPD clinical practice guidelines etc.)	
	13	Report prevention advice, compliance provided and any modification of health-related behavior	
	14	If tooth samples (extracted tooth) were used, include information on the type of tooth, status of development or resorption, and condition of the tooth (non-infectious/infectious/sound)	
	15	Report how success and failure of restorative care was defined from dentist’s, child’s and carer’s point of view

**Table 8:** Checklist items in the “Pulp Therapy” theme

Topic	Item number	Checklist items	Reported on page number
Patient information/unit of analysis	1	Include information on the diagnosis of the condition (irreversible pulpitis, apical periodontitis) and diagnostic method/criteria used on the study	
	2	Include information on the rationale for the treatment performed (pulp capping/partial pulpotomy/ pulpotomy/pulpectomy)	
Intervention	3	Report detailed steps involved in the pulp therapy procedure (anesthesia, isolation, removal of pulp)	
	4	Include rationale for selecting appropriate type of pulp therapy medicament and the final restoration	
	5	For pulp capping and pulpotomy, include information on the exposure site, size, etiology (carious/iatrogenic/trauma), and status of hemostasis (appearance, duration)	
	6	For pulpectomy, include information on the number of visits, root canal anatomy, root length determination, method of canal preparation (hand/rotary), irrigants/intracanal medicament used, obturation material and technique, and quality of obturation	
	7	For lesion sterilization and tissue repair, include information on the type of antibiotics used, instrumentation technique and the timing of placement of final restoration	
Outcomes	8	Include all information on the outcome assessment methods (e.g. clinical, radiographic, histology) and criteria used to define success/failure	
	9	Report specific clinical and radiographical outcomes related to pulp therapy procedure (for example, abscess, internal root resorption etc.)	
	10	Report the time intervals of evaluation or follow-ups	
	11	Report the time period from the start to the end date of the study (minimum 12 months, preferable 24 months or more)	
	12	Report the status of root resorption, presence of first permanent molar, and effect on development of succedaneous tooth (for example, defects, delay in eruption etc.)	
	13	Report if the outcome assessors had knowledge of the clinical and/or radiographic outcomes

**Table 9:** Checklist items in the “Traumatology” theme

Topic	Item number	Checklist items	Reported on page number
Patient information/unit of analysis	1	In reporting a case, report complete details of the trauma incident (when, where, and how)	
	2	Report the level of consciousness using standardized assessment criteria (for example, AVPU Scale, Glasgow Coma Scale etc.) and cranial nerve assessment, where relevant	
	3	Report any suspicion of non-accidental injury	
	4	Report the reason for acquiring a specific diagnostic imaging modality	
	5	If diagnostic images are not presented in the publication, report the findings and the reason for not reporting in the study	
Moderators	6	Report the time taken between the trauma and the provision of dental treatment	
	7	For emergency visits, report duration of waiting, pre-medications, psychological state of child and parent at the time of examination, where relevant	
	8	Report if the child has previously experienced dental trauma. If so, include the dental treatment the child has previously undergone and their level of dental anxiety	
Intervention	9	Include information on the rationale for treatment of specific traumatic dental condition	
	10	Report whether the treatment protocol was followed according to evidence-based guidelines (specific to primary or permanent teeth)	
	11	Report any acute intervention was provided following the trauma, including hemostasis, analgesics etc	
Outcome	12	Report if the trauma resulted in any referrals and subsequent evaluations

**Table 10:** Checklist items in the “Interceptive Orthodontics” theme

Topic	Item number	Checklist items	Reported on page number
Moderators	1	Include information on how the abnormal growth and development of the focus (teeth/jaws) can affect the function and anatomic features of an individual, where relevant	
	2	Include information on the risk factors that can affect or influence the typical growth and development of the focus of the study including the age or stage (primary dentition/mixed dentition/permanent dentition)	
Intervention	3	Include information on the evidence-based treatment used to correct the malocclusion in children and adolescents (for example, intra-oral and extra-oral appliances)	
	4	Include information on a space analysis in mixed dentition and the use of appropriate evidence-based methods, where relevant	
	5	Report all the components of the appliance (wire size, acrylic) along with the commercial details of the product, where relevant	
Outcomes	6	Report child/ caregiver/ parent satisfaction and quality of life on the orthodontic treatment outcomes, where relevant	
	7	Report patient compliance, habits, oral hygiene, and the impact of orthodontic treatment on dental/gingival health	
	8	Report clinical complications in orthodontic or dento-facial orthopedic treatment, if presented	
	9	Report duration of the orthodontic therapy and adverse effects, where relevant	

### Online consensus meeting

A concern was raised regarding one item in the “Pulp Therapy” section corresponding to Item 11 “Report the time period from the start to the end date of the study”. One member indicated that the minimum duration of reporting the outcomes of pulp therapy could be included in this item. Other members specified that studies have been submitted for review with a follow up period of just 6 months after pulp therapy procedure. After a brief discussion, it was agreed that 12 months should be considered as a minimum follow up period to report pulp therapy research. Item 11 was modified accordingly to “Report the time period from the start to the end date of the study (minimum 12 months, preferable 24 months or more)”. All 128 items in the RAPID checklist were unanimously approved by the members at the online meeting.

### Data analysis of the scores

A total of 69 participants responded in the Round 1 Delphi, and 62 in the Round 2 Delphi. The percentage distribution of scores for items in the themes were categorized as Low (Scores 1 to 3), Moderate (Scores 4 to 6), and High (Scores 7 to 9). The percentage for high scores (Scores 7 to 9) ranged from 69.57% to 100% for individual items. The mean percentage for the themes ranged between 78.77% and 89.96% for themes “Interceptive Orthodontics” and “Pulp Therapy” respectively. Similarly, a minimum overall weighted mean score of 7.51 (out of 9) was observed in theme “Interceptive Orthodontics” and a maximum of 8.28 (out of 9) for “Pulp Therapy” (Table 11). One-way ANOVA showed evidence of a statistically significant difference in the overall weighted mean scores between the themes (p < 0.05).

**Table 11:** Weighted mean and percentage distribution of scores between the themes

S. no	Themes	9-point rating Likert scale (%)^a^			Overall Weighted mean^a^	Variance^b^
		1 to 3	4 to 6	7 to 9		
1	General	2.41	12.01	85.58	7.95	0.249
2	Oral Medicine, Pathology and Radiology	0.98	16.99	82.02	7.80	0.104
3	Children with Special Health Care Needs	3.08	17.91	79.01	7.63	0.075
4	Sedation and Hospital Dentistry	1.76	15.44	82.79	7.90	0.084
5	Behavior Guidance	2.24	16.21	81.55	7.78	0.138
6	Dental Caries	4.08	13.21	82.72	7.78	0.109
7	Preventive and Restorative	2.10	11.88	86.02	7.97	0.295
8	Pulp Therapy	1.07	8.98	89.96	8.28	0.154
9	Traumatology	3.40	14.02	82.59	7.82	0.298
10	Interceptive Orthodontics	4.08	17.15	78.77	7.51	0.156

^a^Based on 62 responses at the end of Round 2 Delphi

^b^p < 0.05, one-way ANOVA showing statistically significant difference between the weighted mean scores

## Discussion

A comprehensive list of critical elements that should be used to support completeness of research reporting in Pediatric Dentistry has been developed using a Delphi consensus process. We believe this is the first ever reporting guideline established for a specialty in dentistry. Most of the existing reporting guidelines are based on the study design, for example, randomized trials [17], observational studies [18], systematic reviews [19], animal studies [20], and case reports [21]. Some reporting guidelines have been adapted to a specific specialty or field by integrating the items already presented in the original guideline and by addition of sections relevant to the field, for example, extensions of CONSORT for reporting trials have been adapted for traditional Chinese medicine [22], herbal medicine [23], and acupuncture [24]. Another approach to this process is to exclusively draft the items relevant to the specialty by using the items as a base outline. This approach had been followed for reporting studies in Endodontics, for example, Preferred Reporting Items for Randomized Trials in Endodontics (PRIRATE) [25], and similarly, for other study designs including animal studies (PRIASE) [26], and case reports (PRICE) [27]. Although these provide a comprehensive list of items as a guidance for reporting different study designs in a specialty, the approach was still based on the study design, and not exclusively focused on reporting elements within a specialty. In the current study, a different approach was taken by developing reporting items specific to research in Pediatric Dentistry and focusing on the subject related items without involving the items related to methodological content in study designs.

The themes in the RAPID guideline were broadly classified to represent topics in Pediatric Dentistry. The most challenging task in this project was determining the number of items to be included under each theme as the topics covering the length and breadth of Pediatric Dentistry were exhaustive. The EG identified and vetted the checklist to include only pertinent items, prior to the Delphi process. A total of 151 items was initially included in the Round 1 Delphi. Based on the responses from the RDG members, the items reduced to 128 at the end of Round 2. The RAPID checklist has been categorized into a “General” theme containing items applicable to reporting research across all areas in Pediatric Dentistry. The subsequent themes are specific to the topic of interest, for example, Dental Caries, Pulp Therapy, Traumatology, Interceptive Orthodontics, amongst others. The outcome of this effort is that future authors will be expected to ensure that all the items in the “General” checklist have been reported in their research, in addition to the research theme. This is separate from reporting guidelines recommended for specific study designs. Several journals have endorsed the reporting guidelines for study designs in their “Guidelines to the Authors” and some require a completed reporting checklist along with the submission of the manuscript [28, 29].

It is to be noted that the RAPID statement provides guidance for reporting items specific to Pediatric Dentistry and authors are strongly recommended to concurrently adhere to the reporting guidelines specific to the study design. To ensure adequate reporting practices, we have included an item in the “General” checklist that directs authors to follow the reporting guidelines for study designs (Table 1, Item 1: Indicate that the study adhered to reporting guidelines for main study types (or appropriate extensions where one exists). For example, Case Report—CARE, Observational study—STROBE, Clinical trial- CONSORT, and Systematic review—PRISMA). For the purpose of illustration, authors reporting randomized trials in pulp therapy should follow the reporting items in the “General” checklist (28 items), and the items in the “pulp therapy” checklist (13 items). In addition, items in the Consolidated Statement for Reporting Trials (CONSORT) checklist (37 items) should be included. We strongly believe this approach would enable comprehensive reporting of items relevant to both study design, and specific to Pediatric Dentistry. This would further ensure consistent reporting of research conducted in different parts of the world, facilitating comparison and analysis of global data.

In the current study, the weighted mean has been employed since it takes into account the scores obtained for each item in the Likert 9-point scale and the number of respondents who scored the items [16]. In the protocol publication, we indicated that the median and interquartile ranges would be calculated to analyze the items in the RAPID checklist [15]. Since the data were normally distributed, we employed weighted mean method which is a robust method of assessment. In addition, it should be noted that in the protocol publication, we have indicated that the items would be included if only agreed by 80% of the participants [15]. However, to accommodate additional items for revision in subsequent Delphi rounds, the acceptance rate was reduced to 70% and items scored between 7 and 9 by over 70% of participants were included in the checklist. This acceptance rate was consistent with most of the Delphi studies published in the literature [25–27]. It is to be noted that the RDG members constituted members of healthcare professionals like Pediatric Dentists, General Dentists, Dental Nurse, Academics, as well as parents or caretakers. The variations in the scores could be attributed to the knowledge, attitude, and experience of RDG members in scoring the items in the Likert 9-point scale. No previous consensus development papers have reported analytical data on the outcomes of the Delphi process so we could not compare the results of our study with similar studies published in this area. The initial plan was to have the face-to-face consensus meeting in an international Pediatric Dentistry conference but due to the COVID-19 pandemic restrictions, it was agreed to conduct the meeting through video conferencing. Since the meeting was conducted online, we believe it allowed more members to participate and share their views compared to an in-person participation at a specific location. It is to be noted that the number of participants in the Delphi process were relatively higher than the numbers mentioned in the study protocol with 69 participants in the online Delphi and 25 participants in the face-to-face consensus meeting [15]. However, we did not have the same distribution of participants across different criterion, for example, only 2 child/parents participated in the Delphi compared to 4 hoped for in the child/parent criteria in the study protocol [15], which remains as a limitation of the study.

### Future directions

#### Explanation and elaboration document

The EG will prepare a detailed explanation and elaboration document focusing on the items in the RAPID statement. This will help guide readers as to the specific information to be reported under each item.

#### Endorsement

Steps will be taken for the RAPID statement to be endorsed by various reporting guideline organizations, including EQUATOR (enhancing the quality and transparency of health research). The RAPID project development protocol has already been endorsed by the EQUATOR network [30]. Additionally, editors of journals publishing research in Pediatric Dentistry, Pediatric Dental academies and societies, research and government organizations will be contacted to request endorsement of the RAPID statement. The importance of accurate reporting will be emphasized by introducing the RAPID statement in the research methodology modules of pre-doctoral and post-doctoral courses in Pediatric Dentistry.

#### Translation

The RAPID statement will be translated to various international languages for maximum reachability and use.

#### Website

An exclusive website for the RAPID project has been developed and can be accessed at www.rapid-statement.org. Information regarding the members, resources, and contact information has been provided on the website. Members of the public and stakeholders can provide their feedback through the website. The EG will maintain the website, and any revisions regarding the project will be updated periodically.

## Conclusions

The items presented in the RAPID statement under specific themes in Pediatric Dentistry have been developed through a consensus process by an expert panel. This will provide guidance to authors, reviewers, and editors to ensure all elements have been adequately reported relevant to the study. Adherence to the RAPID guidelines would enable transparent and accurate reporting of high-quality research in Pediatric Dentistry that benefits patients, clinicians, researchers, and other stakeholders involved in the care and well-being of children.

## Supplementary Information

**Additional file 1.** Checklist of the Conducting and Reporting of Delphi Studies (CREDES).

## Data Availability

The datasets used and/or analyzed during the current study are available from the corresponding author on reasonable request from Dr. Jayakumar Jayaraman or Dr. Vineet Dhar.

## Abbreviations

RAPID: Reporting stAndards for research in PedIatric Dentistry
RDG: RAPID Delphi Group
EG: Executive Group
EBP: Evidence-based practice
CREDES: Conducting and reporting Delphi study
CONSORT: Consolidated Standards of Reporting Trials
STROBE: Strengthening the reporting of observational studies in epidemiology
PRISMA: Preferred reporting items for systematic reviews and meta-analyses
CARE: Case reports
EQUATOR: Enhancing the quality and transparency of health research
