# Supplementary material for: Reporting stAndards for research in PedIatric Dentistry (RAPID): an expert consensus-based statement
Source: BMC Oral Health. 2021 Jul 23;21:369. doi: 10.1186/s12903-021-01698-7 (PMC8299173; doi:10.1186/s12903-021-01698-7)
Supplement: Supplementary file 1 — Additional file 1. Checklist of the Conducting and Reporting of Delphi Studies (CREDES). [file 12903_2021_1698_MOESM1_ESM.docx]

**Supplementary Table:** Submission of Checklist of the Conducting and Reporting of Delphi Studies (CREDES)

| **Topics** | **Items** | **Reported in Page** |
| --- | --- | --- |
| **Rationale for the choice of the Delphi technique** | Justification | 2 |
| **Planning and design** | Planning and process | 3 |
|  | Definition of consensus | 4 |
| **Study conduct** | Information input | 5 |
|  | Prevention of bias | 5 |
|  | Interpretation and processing of results | 6 |
|  | External validation | 6 |
| **Reporting** | Purpose and rationale | 3 |
|  | Expert panel | 4 |
|  | Description of the methods | 4,5 |
|  | Procedure | 5,6 |
|  | Definition and attainment of consensus | 7 |
|  | Results | 7,8 |
|  | Discussion of limitations | 9,10 |
|  | Adequacy of conclusions | 11 |
|  | Publication and dissemination | 10,11 |
